# Dynamic analysis of MR-PET data on brain tumors

**DOI:** 10.1186/2197-7364-1-S1-A56

**Published:** 2014-07-29

**Authors:** Ana Morgado, Liliana Caldeira, Nuno da Silva, C Filss, N Matela, K-J Langen, N Jon Shah

**Affiliations:** Institute of Biophysics and Biomedical Engineering, Faculty of Sciences, University of Lisbon, Kragujevac, Portugal; Institute of Neuroscience and Medicine – 4, Forschungszentrum Jülich, Kragujevac, Germany

The introduction of hybrid MR-PET scanners offers new perspectives to better correlate MR and PET data with respect to time and space domain. In case of brain tumor patients, dynamic susceptibility contrast (*DSC*)-*MRI* is often used to measure perfusion levels of brain, while dynamic [^18^F]-fluoro-ethyl-tyrosine (FET)-PET provides additional functional information.

The dynamic analysis of data is becoming more relevant, rather than static analysis, and with this the extraction of parametric images. In this context, the aim of this work is to compare dynamic MR-PET data based on different features.

A FET-PET scan was carried out on the 3T-MR-BrainPET [1] on each patient. Data was acquired for 60 min after injection in list-mode and reconstructed using OSEM3D software (4 subsets, 32 iterations). All MRI sequences were acquired simultaneous to the PET measurement. In this work, MPRAGE and DSC-MRI based on an EPI sequence images are used.

For the dynamic analysis, data was co-registered and the same parameters were extracted, with Matlab, from MR-PET data to produce peak, time to peak (TTP), area under the curve (AUC) and wash-in parametric images.

**Figure 1:**
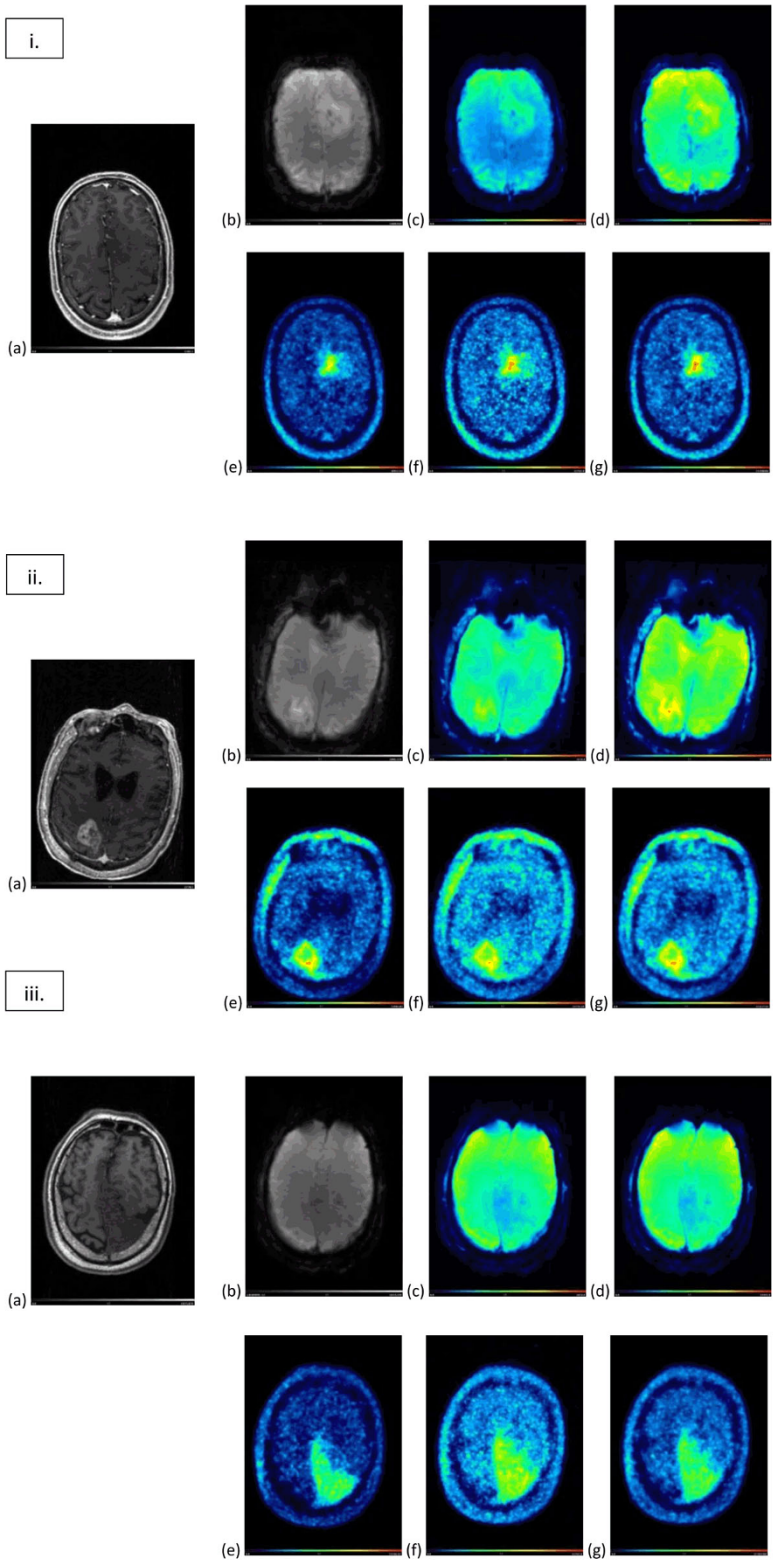
Analyzed datasets (i, ii and iii). MRI images (a) MPRAGE (post contrast), (b) DSC-MRI EPI, and the corresponding extracted parametric images: (c) peak and (d) AUC. (e) FET-PET image (summed image of 20-40 min p.i., previously filtered with a 2 mm Gaussian filter) and the corresponding extracted parametric images of (f) peak and (g) AUC.

Results from 3 of the analyzed datasets from patients with brain tumors are presented on Figure 1. MPRAGE, DSC-MRI EPI sequence and FET-PET images and the extracted parametric images are shown.

In FET-PET parametric images an uptake area can be identified in the region of morphological changes in anatomical MR. The parametric images provide extra information in addition to the summed FET-PET images.

**Figure 2:**
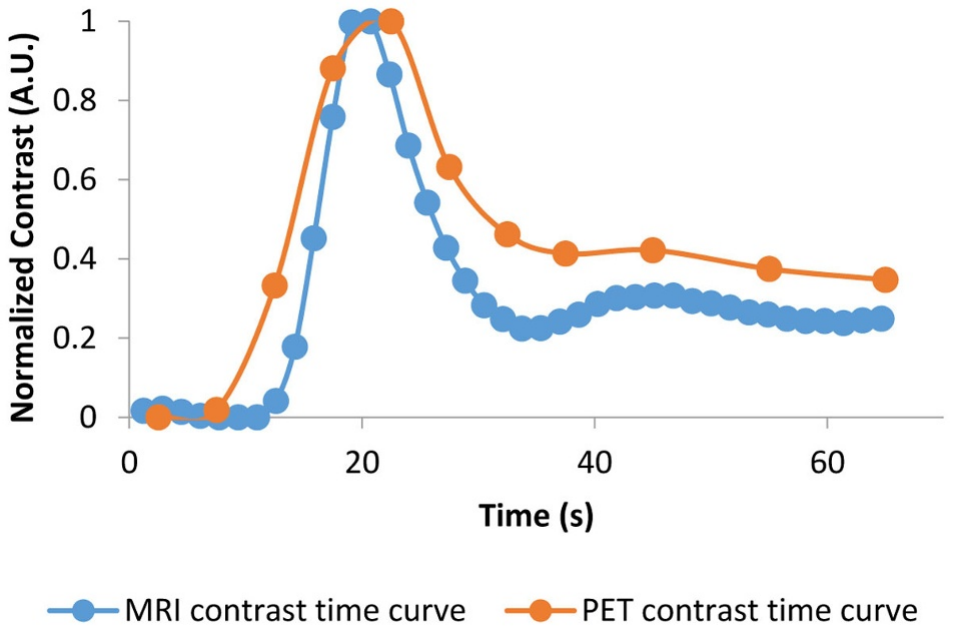
MRI and PET contrast time curves. The FET-PET data was truncated (the total acquisition time was 3600 s) in order to merge the two curves in one graph. The MRI and PET contrast time curves were normalized to the maximum to be able to compare the shape of the two curves. MRI data was converted to C(t) using the formula from [2].

The time curves obtained from a gray-matter region defined on MPRAGE are presented on Figure 2.

The extracted parametric images from dynamic MR-PET showed good spatial and temporal agreement (Figure 1). These results suggest that dynamic MR-PET may have extra information about biology and that the combination of simultaneous acquisition and analysis may be beneficial.
